# Membrane potential and delta pH dependency of reverse electron transport-associated hydrogen peroxide production in brain and heart mitochondria

**DOI:** 10.1007/s10863-018-9766-8

**Published:** 2018-08-17

**Authors:** Tímea Komlódi, Fanni F. Geibl, Matilde Sassani, Attila Ambrus, László Tretter

**Affiliations:** 1 Department of Medical Biochemistry, MTA-SE Laboratory for Neurobiochemistry, Semmelweis University, 37-47 Tűzoltó St, Budapest, 1094 Hungary; 2 Present Address: Department of Neurology, Philipps University Marburg, 35043 Marburg, Germany; 3 Present Address: Department of Neuroscience, Sheffield Institute for Translational Neuroscience (SITraN), University of Sheffield, Sheffield, UK

**Keywords:** Reactive oxygen species, Mitochondria, Proton motive force, Membrane potential, Reverse electron transport, Nigericin, Valinomycin, Succinate, Alpha-glycerophosphate

## Abstract

Succinate-driven reverse electron transport (RET) is one of the main sources of mitochondrial reactive oxygen species (mtROS) in ischemia-reperfusion injury. RET is dependent on mitochondrial membrane potential (Δψ_m_) and transmembrane pH difference (ΔpH), components of the proton motive force (*pmf*); a decrease in Δψ_m_ and/or ΔpH inhibits RET. In this study we aimed to determine which component of the *pmf* displays the more dominant effect on RET-provoked ROS generation in isolated guinea pig brain and heart mitochondria respiring on succinate or α-glycerophosphate (α-GP). Δψ_m_ was detected via safranin fluorescence and a TPP^+^ electrode, the rate of H_2_O_2_ formation was measured by Amplex UltraRed, the intramitochondrial pH (pH_in_) was assessed via BCECF fluorescence. Ionophores were used to dissect the effects of the two components of *pmf*. The K^+^/H^+^ exchanger, nigericin lowered pH_in_ and ΔpH, followed by a compensatory increase in Δψ_m_ that led to an augmented H_2_O_2_ production. Valinomycin, a K^+^ ionophore, at low [K^+^] increased ΔpH and pH_in_, decreased Δψ_m_, which resulted in a decline in H_2_O_2_ formation. It was concluded that Δψ_m_ is dominant over ∆pH in modulating the succinate- and α-GP-evoked RET. The elevation of extramitochondrial pH was accompanied by an enhanced H_2_O_2_ release and a decreased ∆pH. This phenomenon reveals that from the pH component not ∆pH, but rather absolute value of pH has higher impact on the rate of mtROS formation. Minor decrease of Δψ_m_ might be applied as a therapeutic strategy to attenuate RET-driven ROS generation in ischemia-reperfusion injury.

## Introduction

There is a large body of experimental evidence demonstrating pathologically enhanced mitochondrial reactive oxygen species (mtROS) production in several diseases such as diabetes, neurodegenerative conditions including Alzheimer’s and Parkinson’s diseases, diabetes, and ischemia-reperfusion injury; for review see (Beal 1996; Giacco and Brownlee 2010; Chouchani et al. 2014). Respiratory Complex I (CI) is a primary source of mtROS and its dysfunction is thought to be pathologically relevant (Cadenas et al. 1977, Grivennikova and Vinogradov 2006, Treberg et al. 2011). In isolated mitochondria CI-mediated mtROS generation can be initiated under the following conditions: 1) with NADH-linked substrates (such as glutamate and malate), which generate mtROS at a relatively low rate; 2) with NADH-linked substrates in the presence of a CI inhibitor, like rotenone producing high rate of ROS; 3) with FADH_2_-linked substrates like succinate or (Lambert and Brand 2004; Zoccarato et al. 2004; Treberg et al. 2011; Orr et al. 2012) alpha-glycerophosphate (α-GP) (Tretter et al. 2007b, c). In hyperpolarized non-phosphorylating mitochondria, FADH_2_-linked substrates generate mtROS at a higher rate by supporting a reverse electron transport (RET) which occurs from Complex II (CII) or alpha-glycerophosphate dehydrogenase (α-GPDH) to CI via the Q-junction (Treberg et al. 2011). According to prior reports, succinate-driven mtROS production appears to have the highest rate in isolated murine mitochondria in the absence of ADP compared to NADH-linked substrates initiated mtROS (Korshunov et al. 1997; Kwong and Sohal 1998; Votyakova and Reynolds 2001; Liu et al. 2002; Zoccarato et al. 2011); this is attributed primarily to RET towards CI and partially to the forward electron transport (FET) towards CIII (Grivennikova and Vinogradov 2006; Treberg et al. 2011; Zoccarato et al. 2011; Quinlan et al. 2013). Upon succinate oxidation, in the absence of ATP synthesis, FET secures the energy demands of RET.

Rate of RET-associated ROS production is thought to be dependent on *pmf* which comprises mitochondrial transmembrane potential (Δψ_m_) and mitochondrial transmembrane pH gradient (∆pH) (Liu 1997; Votyakova and Reynolds 2001; Lambert and Brand 2004). It is a well-known phenomenon that high *pmf*, such as the one measured in the absence of ADP, is required for maintenance of RET. It has been shown that succinate- or α-GP-fuelled RET is very sensitive to minor changes in Δψ_m_ in isolated mammalian (Tretter and Adam-Vizi 2007) and Drosophila (Miwa and Brand 2003) mitochondria.

More specifically, a 10% decrease in Δψ_m_ (caused by an uncoupler agent) gave rise to a 90% decrease in succinate-driven ROS production in rat heart mitochondria (Korshunov et al. 1997). The other component of *pmf*, ∆pH, also appears to have a regulating effect on mtROS formation. Upon acidification of the matrix, mtROS generation is decelerated, which can be explained by the stabilisation of the semiquinone radicals (SQ^**.**-^) (Selivanov et al. 2008). The question arises as to which component of *pmf* plays the key role in the control of mtROS production. According to Lambert and Brand (Lambert and Brand 2004), succinate-driven ROS production is more dependent on ∆pH than on Δψ_m_, as detected in mitochondria isolated from rat skeletal muscle. On the contrary, Selivanov and co-workers (Selivanov et al. 2008) revealed that mtROS generation is significantly affected by the actual value of pH itself (extramitochondrial pH; pH_extra_ and intramitochondrial pH; pH_in_), and not much influenced by ∆pH or Δψ_m_, as measured in rat brain mitochondria.

The aim of the present study was to clarify which of the two components of *pmf* has a predominant role in the control of mtROS formation and to assess whether absolute pH value modulates RET-dependent mtROS production. We also aimed to test whether the effect of Δψ_m_, ΔpH, and absolute pH values on ROS formation is different in brain compared to heart muscle mitochondria. Δψ_m_ and ΔpH usually change in the same direction; for example, uncoupling depolarisation (decrease of Δψ_m_) is generally followed by a decrease in ∆pH as well. With ionophores, like valinomycin and nigericin, it is possible to dissect the two components of *pmf*: Δψ_m_ and ΔpH can be varied in a different direction. Nigericin decreases pH_in_ (Rottenberg and Lee 1975) and hyperpolarises Δψ_m_ (Selivanov et al. 2008), whilst valinomycin elevates pH_in_ (Selivanov et al. 2008) and depolarizes Δψ_m_ (Selivanov et al. 2008) under specific conditions. In the present study, Δψ_m_, pH_in_, and H_2_O_2_ production were measured systematically and ∆pH was calculated. To scrutinize Selivanov’s theory, pH dependence of the above-mentioned parameters was examined. In contrast to Lambert and co-workers (Lambert and Brand 2004), we concluded that the succinate-driven RET-evoked ROS production is more dependent on Δψ_m_ and less influenced by ∆pH in both guinea pig brain and heart mitochondria. Furthermore, we showed, in agreement with Selivanov and colleagues, that absolute pH rather than ∆pH itself modulates succinate- and α-GP-driven RET. Our results suggest that lowering Δψ_m_ might be an effective solution to reduce the RET-provoked mtROS load in conditions like ischemia-reperfusion where oxidative stress and high Δψ_m_ prevail.

## Materials and methods

### Chemicals

Standard laboratory reagents, except ADP, were obtained from Sigma (St. Louis, MO, USA). ADP was purchased from Merck (Darmstadt, Germany). BCECF/AM and Amplex UltraRed were obtained from TermoFisher Scientific (Waltham, MA, USA).

### Preparation of mitochondria

Mitochondria were prepared from albino guinea pig brain cortex using a Percoll gradient (Rosenthal et al. 1987,Tretter and Adam-Vizi 2007) and from whole heart using differential centrifugation (Mela and Seitz 1979,Korshunov et al. 1997), as previously described. Animal experiments were performed in accordance with the Guidelines for Animal Experiments of Semmelweis University. A modified biuret method was used to determine mitochondrial protein concentration (Bradford 1976).

#### Brain mitochondria

The brain was rapidly homogenized in Buffer A (in mM: 225 mannitol, 75 sucrose, 5 HEPES, 1 EGTA, pH 7.4) and centrifuged for 3 min at 1300 *g*. The supernatant was centrifuged for 10 min at 20,000 *g*, and the resulting pellet was resuspended in 15% Percoll and layered on a discontinuous gradient consisting of 40 and 23% Percoll. This was centrifuged for 8 min at 30,700 *g* using no brake. After resuspension of the lower fraction in Buffer A, centrifugation was applied at 16,600 *g* for 10 min. Pellet was resuspended in Buffer A and centrifuged at 6300 *g* for 10 min. Subsequently, supernatant was discharged, and the pellet was resuspended in Buffer B (in mM: 225 mannitol, 75 sucrose, 5 HEPES, pH 7.4). All operations above were performed either on ice or at 4 °C (Komary et al. 2008).

#### Heart mitochondria

Mitochondria from heart were isolated following the modified protocol of Korshunov and co-workers (Korshunov et al. 1997). The heart was repeatedly washed in homogenisation buffer (in mM: 200 mannitol, 50 sucrose, 5 NaCl, 5 MOPS, 1 EGTA, 0.1% BSA, pH 7.15) to remove residual blood. Afterwards, it was cut into small pieces with scissors under 2.5 ml homogenisation buffer supplemented with 10 U protease (Protease from Bacillus licheniformis, Type VIII). After adding 17 ml of homogenisation buffer, the preparation was properly homogenised and centrifuged for 10 min at 10,500 *g*. The supernatant was discharged, the pellet was resuspended in 25 ml homogenisation buffer, and then centrifuged for 10 min at 3000 *g*. The supernatant was centrifuged for 10 min at 10,500 *g* and the formed pellet was resuspended in the homogenisation buffer. All operations above were performed either at 4 °C or on ice.

### Buffers

Depending on the requirement of K^+^ of the applied ionophore (nigericin or valinomycin), one of the following media was applied in the relevant experiments:

Standard medium *A* (high K^+^ content for nigericin; in mM): 125 KCl, 20 HEPES, 2 KH_2_PO_4_, 0.1 EGTA, 1 MgCl_2_, and 0.025% BSA.

Standard medium *B* (low K^+^ content for valinomycin to avoid mitochondrial swelling; in mM): 240 sacharose, 10 Tris, 2 KH_2_PO_4_, 4 KCl, 0.1 EGTA, 1 MgCl_2_, and 0.025% BSA. pH of the respiratory media was adjusted prior to the measurements, in the absence of mitochondria, with HCl or NaOH to 6.4, 6.8, 7.0, 7.2, 7.4, 7.6 or 8.0. Addition of mitochondria suspended in buffered solution and addition of high concentrations of respiratory substrates (succinate or α-GP) could shift the pH of the incubation medium slightly. In order to calculate an accurate ∆pH, pH_extra_ measured in the presence of mitochondria and respiratory substrate was applied in this study.

### Measurement of mitochondrial H_2_O_2_ production

The assay is based on detection of H_2_O_2_ in the medium using the Amplex UltraRed fluorescent dye. In the presence of horseradish peroxidase (HRP), Amplex UltraRed reacts with H_2_O_2_ in a 1:1 stoichiometry producing fluorescent Amplex UltroxRed. HRP (5 U/2 ml) and Amplex UltraRed (3 μM) were added to standard medium *A* or *B*. Subsequently, mitochondria (0.05 mg/ml) and succinate (5 mM) or α-GP (20 mM) were added. Resorufin fluorescence was detected using a Photon Technology International (PTI; Lawrenceville, NJ, USA) Deltascan fluorescence spectrophotometer. The excitation wavelength was 550 nm, while the emission was detected at 585 nm. At the end of each experiment, the fluorescence signal was calibrated with 100 pmol H_2_O_2_. All the measurements were performed at 37 °C.

### Measurement of the mitochondrial membrane potential (Δψ_m_)

#### Measurement with safranine-O

Δψ_m_ was assessed using safranine-O, a lipophilic cationic fluorescent dye, which accumulates in the mitochondrial membrane upon hyperpolarisation resulting in fluorescence quenching (Akerman and Wikstrom 1976). Safranine (2 μM) fluorescence (495 nm for excitation, 585 nm for emission) was detected using a Hitachi F-4500 spectrofluorimeter (Hitachi High Technologies, Maidenhead, UK). All measurements were carried out at 37 °C in standard medium *A* or *B*, as previously described.

#### Measurement with TPP^+^ electrode

Δψ_m_ was estimated via the distribution of the tetraphenylphosphonium ion (TPP^+^). TPP^+^ was detected using a custom-made TPP^+^-selective electrode (Kamo et al. 1979), as described previously (Tretter et al. 2007a). Δψ_m_ was calculated using the Nernst equation and the reported binding correction factor for brain mitochondria, as previously described (Rottenberg 1984; Rolfe et al. 1994). The calculation was performed according to Rottenberg and co-workers (Rottenberg 1984) assuming that the matrix volume of the mitochondria is 1 μl/mg protein (D.G. Nicholls, personal communication). The sensitivity of the TPP^+^ electrode was found to be decreased at low Δψ_m_ (less than ~120 mV) (Starkov and Fiskum 2003).

### Measurement of the intramitochondrial pH (pH_in_)

pH_in_ of isolated mitochondria was measured with the acetoxymethyl ester form of 2,7-biscarboxyethyl-5(6)-carboxyfluorescein (BCECF/AM) (Jung et al. 1989), as described earlier (Sipos et al. 2005). Briefly; 100 μl mitochondria (35–40 mg/ml protein) were incubated with 50 μM BCECF/AM in Buffer C (in mM: 225 mannitol, 75 sucrose, 5 HEPES, 0.1 EGTA, pH 7.4) for 10 min at 25 °C. Ice-cold Buffer C (325 μl) was supplemented with 0.1 mM ADP (in order to prevent permeability transition pore opening). Loaded mitochondria were centrifuged for 2 min at 13000 *g*, the supernatant was removed, the pellet was resuspended in 450 μl Buffer C, and this was centrifuged for 2 min at 13000 *g*. The new pellet was resuspended in 450 μl Buffer C *minus* ADP, left standing for hydrolysis (10 min), and then centrifuged for 2 min at 13000 *g*. All centrifugation steps were performed at 4 °C. The supernatant was discharged. The pellet was supplemented with 13 μl Buffer C. BCECF-loaded mitochondria were used within 90 min. For fluorescence measurements, 3 μl aliquots of mitochondria were diluted in 2 ml of standard medium *A* or *B*. Fluorescence ratios were determined using the PTI Deltascan fluorescence spectrophotometer (440 or 505 nm for excitation, 540 nm for emission). Leaching of BCECF from mitochondria was determined by measuring the fluorescence of the supernatant of the centrifuged loaded mitochondria. Corrections were made by subtracting the fluorescence values of the supernatant from those of the experimental values. For calibration, the external and internal [H^+^] were equilibrated at varying pH_extra_ values by the addition of a mixture of 8 μM nigericin (K^+^/H^+^ antiporter), 2.5 μM gramicidin (Na^+^/K^+^ ionophore), and 8 μM monensin (Na^+^/H^+^ antiporter), as previously described (Sipos et al. 2005).

### Statistical analysis

The statistical differences in multiple comparisons were evaluated with ANOVA (SigmaPlot™, Version 11, Systat Software, Inc., San Jose, CA, USA). Values of *p* < 0.05 were considered to be statistically significant.

## Results

In order to dissect Δψ_m_ and ΔpH, the two components of *pmf*, ionophores were introduced throughout the experiments. The standard media *A* contained 2 mM K_2_HPO_4_ and 125 mM KCl*,* whilst standard medium *B* was supplemented with 2 mM K_2_HPO_4_ and 4 mM K^+^. ADP was absent providing a high Δψ_m_ to support RET in succinate- or α-GP-energised mitochondria. At the end of each experiment the uncoupler FCCP was given to eliminate any Δψ_m_ and abolish the succinate- or α-GP-driven RET.

### Effects of nigericin on pH_in_, ΔpH, Δψ_m_, and mtROS production in brain mitochondria at medium pH 7.0

**Fig. 1:**
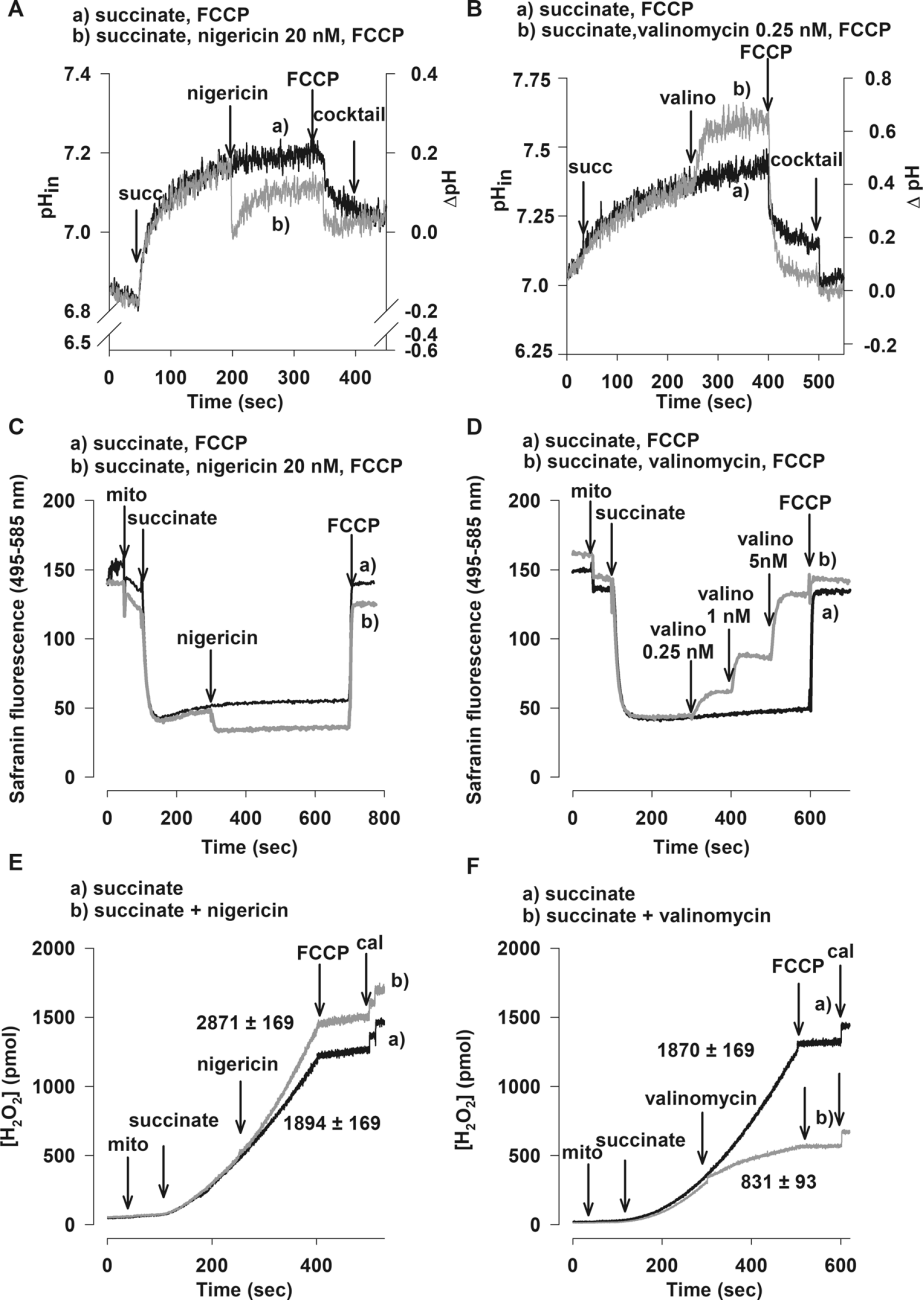
Effect of nigericin (**a, c, e**) and valinomycin (**b, d, f**) on pH_in_ and ∆pH (**a**, **b**), Δψ_m_ (**c**, **d**) and the rate of H_2_O_2_ production (**e**, **f**) in succinate-energised brain mitochondria. Mitochondria (0.05 or 0.1 mg/ml) were incubated in different standard media as described under Materials and Methods. Succinate (5 mM), FCCP (250 nM), valinomycin (0.25 nM), nigericin (20 nM) and cocktail (gramicidin, monensin, nigericin) were given as indicated. ∆pH (**a**, **b**) values were calculated from the difference between pH_in_ and pH_extra_. In *A* and *B* each experiment was calibrated by KOH. In *E* and *F* results (slope) are expressed in pmol/min/mg protein and each experiment was calibrated by 100 pmol H_2_O_2_. For (**a**, **b**, **c**, **d**, **e**, **f**) traces are representative of at least three independent experiments

Nigericin, a K^+^/H^+^ antiporter, allows the electroneutral transport of these two ions in opposite directions across the mitochondrial inner membrane following the K^+^ concentration gradient (Henderson et al. 1969; Rottenberg and Lee 1975). As displayed in Fig. 1, nigericin (20 nM) decreased pH_in_ (Fig. 1a) at pH_extra_ = 6.84 ± 0.01 (medium pH = 7.0) by 0.13 ± 0.04 pH unit and ∆pH from 0.23 ± 0.06 to 0.089 ± 0.02 (Fig. 1c). In addition, nigericin increased Δψ_m_ by 7.78 ± 2.5 mV; Δψ_m_ could not be increased any further by subsequent additions of nigericin. In contrast to Lambert and co-workers (Lambert and Brand 2004), we found that nigericin increased the rate of H_2_O_2_ generation by 52 ± 11% (from 1894 ± 169 to 2871 ± 169 pmol/min/mg protein) in succinate-respiring brain mitochondria (Fig. 1e). We can conclude that in succinate-supported mitochondria, nigericin decreased ∆pH and induced mitochondrial hyperpolarization, simultaneously elevating H_2_O_2_ production.

**Fig. 2:**
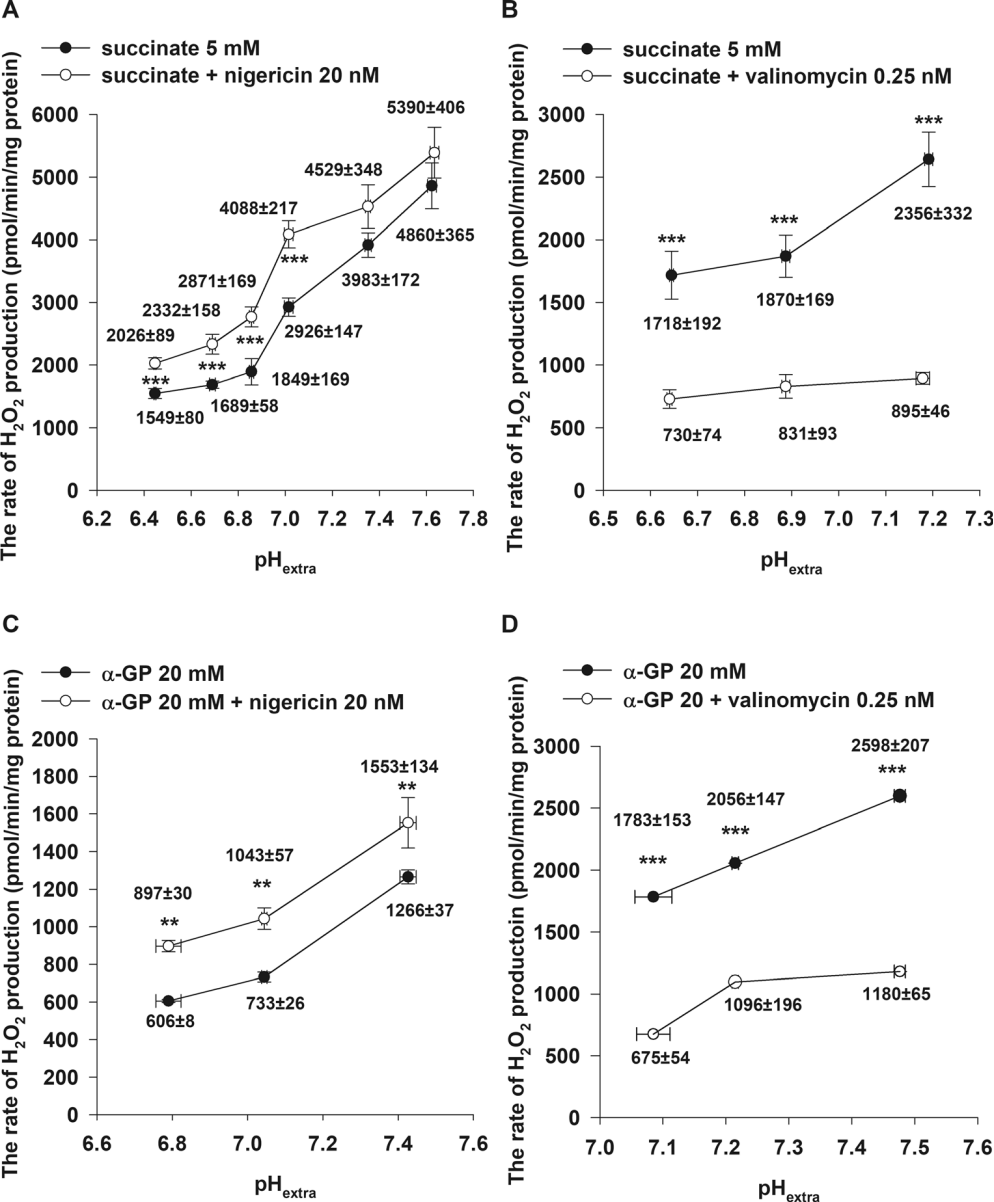
Effect of nigericin (**a, c**) and valinomycin (**b, d**) on the rate of succinate (**a**, **b**) and α-glycerophosphate (**c**, **d**)-driven H_2_O_2_ production as a function of pH_extra_ in brain mitochondria. Mitochondria (0.05 mg/ml) were incubated in the standard media as described under Materials and Methods. Succinate (5 mM), α-glycerophosphate (α-GP; 20 mM), valinomycin (0.25 nM) and nigericin (20 nM) were added. The results are expressed as the rate of H_2_O_2_ production in pmol/min/mg protein mean ± SEM (*n* > 4) and pH_extra_ given as mean ± SEM (n > 4) and written in the graphs; ***p < 0.001; **p < 0.01

In order to gain a deeper insight into the effects of nigericin on RET, α-GP was also applied as a respiratory substrate. Unlike succinate, α-GP does not enter the mitochondria, it is oxidized by α-GPDH on the outer surface of the inner mitochondrial membrane and does not form NADH. Addition of rotenone diminished the H_2_O_2_ production both in succinate and α-GP energised mitochondria, which points to a CI-related ROS production, likely RET (Votyakova and Reynolds 2001). Both respiratory substrates upon their oxidation by succinate dehydrogenase (SDH) or α-GPDH reduce the coenzyme Q (Q; ubiquinone)-junction bypassing CI. Similarly to that observed with succinate, nigericin decreased pH_in_, increased Δψ_m_ (data not shown), and stimulated H_2_O_2_ production (Fig. 2c) in α-GP-energised mitochondria as well.

### Effects of valinomycin on pH_in_, ΔpH, Δψ_m_, and mtROS production in brain mitochondria at pH 7.0

**Fig. 3:**
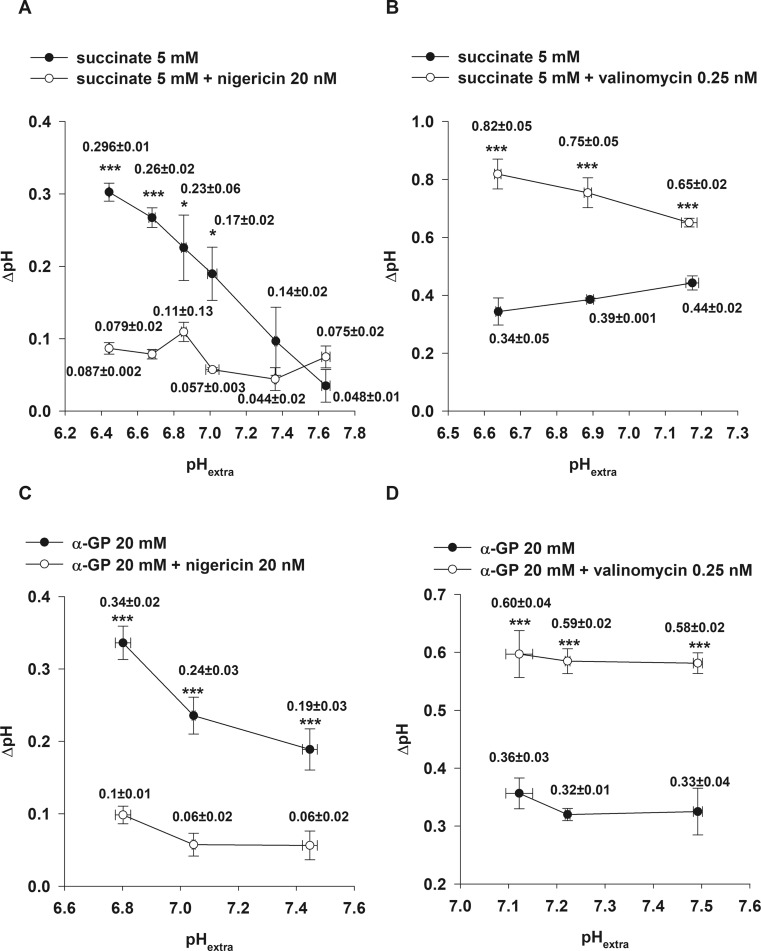
Effect of nigericin (**a, c**) and valinomycin (**b, d**) on ∆pH in succinate (**a**, **b**) and α-glycerophosphate (**c, d**)-respiring brain mitochondria as a function of pH_extra_. Mitochondria were incubated in the standard media as described under Materials and Methods. Succinate (5 mM), α-glycerophosphate (α-GP; 20 mM), valinomycin (0.25 nM) and nigericin (20 nM) were used. The results are expressed as pH value mean ± SEM (n > 4) and written in the graphs; ***p < 0.001; *p < 0.05

Valinomycin is a K^+^ ionophore transporting K^+^ along its electrochemical gradient across the mitochondrial inner membrane. In succinate-supported mitochondria, valinomycin (0.25 nM) increased pH_in_ by 0.38 ± 0.04 pH unit (Fig. 1b)_,_ ∆pH from 0.39 ± 0.001 to 0.75 ± 0.04, and depolarized ∆ψ_m_ in a dose-dependent manner (Fig. 1d). We found that valinomycin decreased the rate of H_2_O_2_ generation by 44.5 ± 4% when mitochondria were supported by succinate (Fig. 1f, trace b). Valinomycin displayed similar effects on α-GP-respiring brain mitochondria. At pH_extra_ = 7.22 ± 0.01 (Fig. 2d), valinomycin alkalized the mitochondrial matrix by 0.26 ± 0.02 pH unit, while ∆pH was increased from 0.32 ± 0.01 to 0.59 ± 0.02 (Fig. 3d) with α-GP. Simultaneously, a decreased rate of the α-GP-evoked H_2_O_2_ production (by 45 ± 14%) was measured, similarly to that observed in succinate-supported mitochondria.

### Effects of pH_extra_ on H_2_O_2_ production, pH_in_, ΔpH, and Δψ_m_ in succinate- and α-GP-respiring brain mitochondria

To examine the influence of changes in pH_extra_ on ∆pH and H_2_O_2_ production, experiments were carried out in standard media *A* (nigericin) or *B* (valinomycin) varying pH from 6.4 to 8.0 (see Materials and Methods).

#### H_2_O_2_ production

As seen in Fig. 2, upon increasing pH_extra_ a sharp increase of succinate- and α-GP-related H_2_O_2_ generation was observed both in the absence *(*Fig. 2a, c*, black circles)* and presence *(*Fig. 2a, c*, white circles)* of nigericin. The nigericin treatment of succinate-supported mitochondria elevated the rate of H_2_O_2_ production significantly between pH_extra_ = 6.45 ± 0.004 and 7.03 ± 0.02 (Fig. 2a)*.* Similarly, in α-GP-respiring mitochondria nigericin increased the rate of H_2_O_2_ formation by 48 ± 3% at pH_extra_ = 6.81 ± 0.01, 42 ± 4% at pH_extra_ = 7.05 ± 0.05, and 21 ± 11% at pH_extra_ = 7.45 ± 0.02 (Fig. 2c). The addition of valinomycin to succinate- and α-GP-supported mitochondria significantly reduced the rate of H_2_O_2_ formation at all different pH_extra_ values (Fig. 2b, d)*.*

#### Δψ_m_

Measuring Δψ_m_ by a TPP^+^ electrode, it was concluded that nigericin always increased the Δψ_m_ approximately to the same level (~ − 195 ˗ -200 mV), even at different pH_extra_ values in brain mitochondria. At pH_extra_ = 6.45 ± 0.004, nigericin hyperpolarized the membrane by 12.5 mV, at pH_extra_ = 6.84 ± 0.013 by 19 mV, and at pH_extra_ = 7.30 ± 0.047 by 8.5 mV. Taken together, these data show that Δψ_m_ and the rate of H_2_O_2_ production were the highest when nigericin was present and the medium was the most alkaline.

#### pH_in_ and ΔpH

As shown in Fig. 3, upon elevation of pH_extra_, ΔpH was concomitantly decreased in both succinate- and α-GP-respiring mitochondria. The addition of nigericin was followed by acidification of the mitochondrial matrix, resulting in a drop of ∆pH (Fig. 3a, c). At the most alkaline pH_extra_ (7.45 ± 0.02), in the presence of succinate, nigericin could neither decrease pH_in_ nor ∆pH. However, in α-GP-respiring mitochondria, nigericin reduced both pH_in_ and ∆pH at all measured pH_extra_ values (Fig. 3c)*.* Valinomycin treatment of both succinate- and α-GP-respiring brain mitochondria caused alkalinization of the mitochondrial matrix and a corresponding elevation of ΔpH (Fig. 3b, d).

### Heart mitochondria. Effects of nigericin and valinomycin on mitochondrial parameters

Detecting RET is also relevant in organs other than the brain, like heart, regarding their exposure to oxidative stress under pathological conditions, like ischemia-reperfusion (Chouchani et al. 2014). To deepen our understanding on RET in heart mitochondria, effects of ∆pH and Δψ_m_ on succinate-supported H_2_O_2_ production were investigated applying the above-mentioned ionophores.

**Fig. 4:**
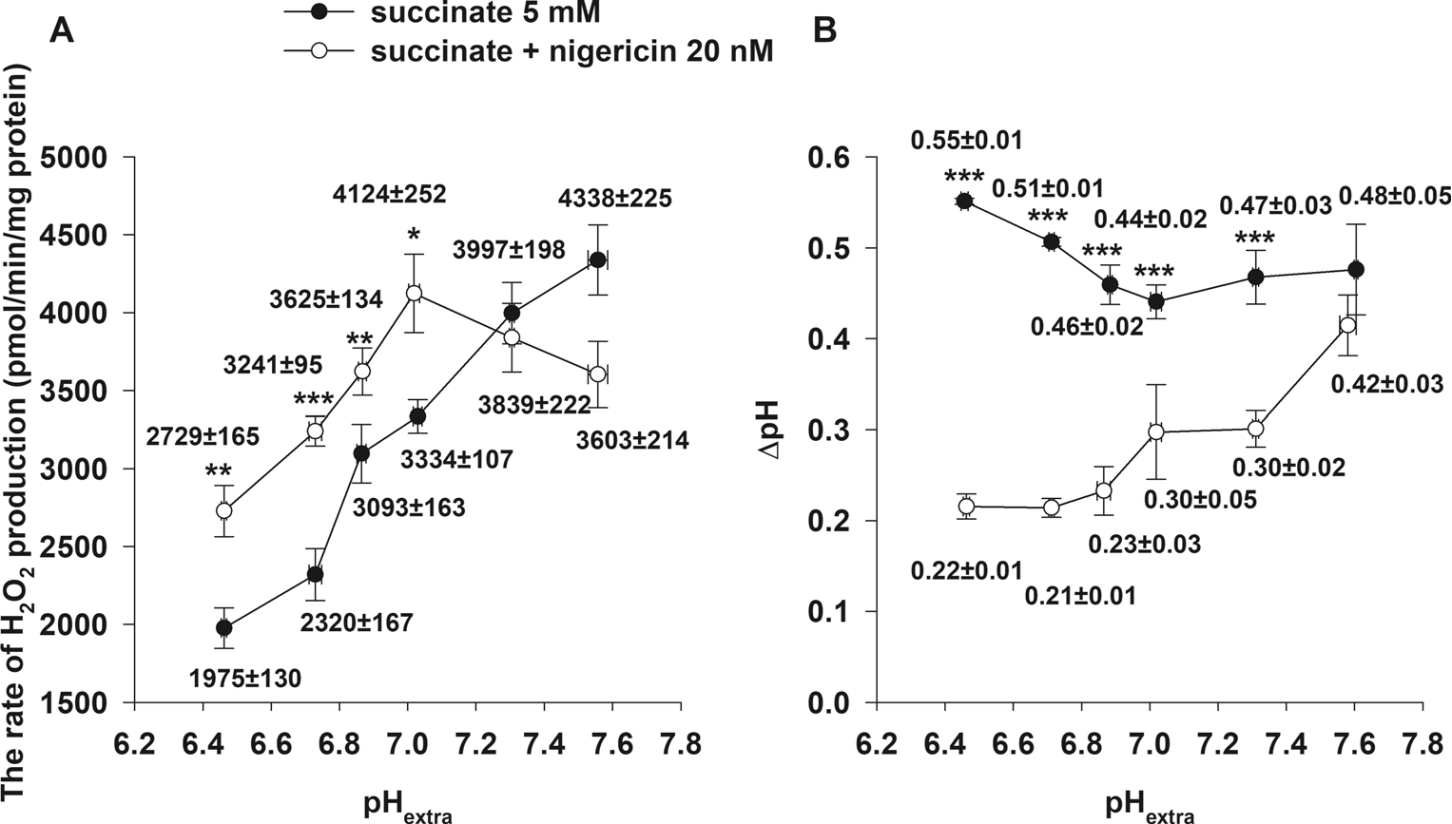
Effect of nigericin on the rate of H_2_O_2_ production (**a**) and on ∆pH (**b**) at different pH_extra_ in succinate-respiring isolated heart mitochondria. Mitochondria (0.05 or 0.1 mg/ml) were incubated in standard medium *A* as described under Materials and Methods. Succinate (5 mM) and nigericin (20 nM) were given. The results of *A* are expressed as the rate of H_2_O_2_ production in pmol/min/mg protein mean ± SEM (n > 4) and written in the graphs, pH_extra_ given as mean ± SEM (n > 4). For *B* the results are expressed in pH values mean ± SEM and written in the graphs (n > 4); ***p < 0.001; **p < 0.01; *p < 0.05

Similarly to brain, in heart mitochondria nigericin hyperpolarized the membrane at various pH_extra_ values. In the absence of nigericin, Δψ_m_ of succinate-supported, non-phosphorylating mitochondria was similar at all pH_extra_ values analogously to brain. In contrast to that observed in brain mitochondria, in heart, the addition of nigericin led to an increase of the rate of succinate-evoked H_2_O_2_ generation only between pH_extra_ = 6.46 ± 0.005 and 7.03 ± 0.008 (Fig. 4a). At a more alkaline pH (pH_extra_ = 7.54 ± 0.002), nigericin decreased the rate of H_2_O_2_ formation by 22 ± 8% *(*Fig. 4a*, white circles)*. In the absence of nigericin, upon elevation of pH_extra_, the rate of the succinate-initiated H_2_O_2_ generation was steeply increasing *(*Fig. 4a*, black circles)*. In the absence of nigericin, ∆pH decreased with incrementing pH_extra_ (pH_extra_ from 6.46 ± 0.005 to 7.03 ± 0.008) until pH_extra_ 7.03 ± 0.008; at pH_extra_ above such value_,_ ∆pH increased *(*Fig. 4b*, black circles)*. Contrary to this, in the presence of nigericin, ∆pH was slightly ascending upon pH_extra_ elevation *(*Fig. 4b*, white circles)* and at pH_extra_ = 7.54 ± 0.002 there was no statistically significant difference between ∆pH in the presence of nigericin compared to ∆pH in its absence.

## Discussion

There is a lack of consensus regarding the role of ∆pH and Δψ_m_ on mtROS generation (Lambert and Brand 2004; Selivanov et al. 2008), therefore, in our study, we aimed to clarify the dependence of succinate- and α-GP-driven H_2_O_2_ production on components of *pmf*. The results presented above allow the conclusion that Δψ_m_ displays a stronger influence on the succinate- or α-GP-supported, RET-initiated H_2_O_2_ production than ΔpH. In this study, we did not only measure H_2_O_2_ production and Δψ_m_, but we also detected matrix pH (pH_in_) with the fluorescent dye BCECF and calculated ΔpH. Under most physiological conditions depolarization of the inner membrane (decrease of the absolute value of Δψ_m_) is associated with a decrease of ∆pH and an elevation of matrix [H^+^]. It is unfeasible to create conditions where one of the components of *pmf* is maintained constant whilst the other one is independently altered. With ionophores however, these two parameters can be changed in opposite directions. In order to increase Δψ_m_, nigericin was applied, which decreased ∆pH and increased H_2_O_2_ production (Fig.1) suggesting that mtROS production is directly proportional to Δψ_m_. If ∆pH was the dominant factor of the RET-initiated H_2_O_2_ formation, then H_2_O_2_ production should have been decreased. To increase ∆pH, valinomycin was added, which simultaneously depolarized the inner membrane and decreased the rate of H_2_O_2_ generation which changed in accordance with Δψ_m_ values. If ∆pH had been the major player in H_2_O_2_ production, then H_2_O_2_ production should have been higher in the presence of valinomycin than in its absence.

Our measurements were carried out not only in brain but also in heart mitochondria, both displaying similar effects. Based on these observations, we can exclude the tissue specific modification of RET– supported H_2_O_2_ generation in these tissues.

In summary, our studies with the two ionophores showed that RET-evoked H_2_O_2_ production always varied in accordance with changes of Δψ_m_, which leads to the conclusion that Δψ_m_ has a greater influence on mitochondrial RET-initiated H_2_O_2_ formation than ΔpH. In addition, we also showed that elevation of pH_extra_ resulted in increased H_2_O_2_ generation, a finding that suggests a clear correlation between absolute pH and H_2_O_2_ production.

### Nigericin

Nigericin, as a K^+^/H^+^ antiporter, is responsible for the electroneutral exchange of K^+^ and H^+^ (Henderson et al. 1969; Bernardi 1999). In our preliminary experiments, the dose-dependent effects of nigericin on Δψ_m_ were studied, and the lowest possible concentration was used which created a maximal mitochondrial hyperpolarization measured by safranin fluorescence (data not shown). Contrary, Lambert and colleagues (Lambert and Brand 2004) as well as Selivanov’s group (Selivanov et al. 2008) applied 100 nM nigericin, which in our hands did neither increase Δψ_m_ further, nor dissipate ΔpH completely but established a new equilibrium with lower pH_in_. ΔpH, after administration of 100 nM nigericin, could be decreased further by addition of 250 nM FCCP and mixture of ionophores (see Materials and Methods). To eliminate confounding factors that could have influenced ROS production (e.g. succinate transport or further metabolism of succinate in the tricarboxylic acid cycle), not only succinate, but also α-GP was used to energize mitochondria and support RET-mediated ROS production. Results with α-GP were qualitatively equivalent to those obtained in succinate-supported mitochondria (Figs. 2 and 3). The stimulating effect of nigericin on H_2_O_2_ generation was more pronounced at acidic pH_extra_. Interestingly, in heart mitochondria, at alkaline pH_extra_, nigericin decreased the rate of H_2_O_2_ release (Fig. 4a). It appears that in heart mitochondria, the diminution in the rate of H_2_O_2_ production at alkaline pH cannot be explained by depolarisation of the mitochondrial membrane.

### Valinomycin

In the presence of valinomycin, the mitochondrial membrane is permeable to K^+^; its effect is highly dependent on the K^+^ concentration of the medium and the applied valinomycin concentration. High K^+^ concentrations in the presence of 2 mM KH_2_PO_4_ and valinomycin lead to high amplitude mitochondrial swelling (Ligeti and Fonyo 1977; Bernardi 1999), therefore, 4 mM KCl was used in valinomycin experiments. It is well known that in isolated mitochondria the highest ΔpH can be achieved at low K^+^ concentration (Mitchell and Moyle 1968; Nicholls 1974; Nicholls 2005). It is noteworthy that ΔpH in low K^+^ medium is about 0.6–0.8 pH unit, but at high K^+^ medium it is only 0.3 pH unit. In our experiments valinomycin caused matrix alkalization and concomitant ΔpH elevation. This observation can be explained by the fact that the valinomycin-induced entry of K^+^ into the mitochondrial matrix usually triggers H^+^ extrusion and P_i_/OH^−^ exchange (Garlid and Paucek 2003). The H^+^ extrusion generally mediates a compensatory decrease in Δψ_m_ and an elevation of respiration both in the succinate- or α-GP-supported mitochondria. Valinomycin-caused depolarisation led to inhibition of RET-supported H_2_O_2_ production.

### Effects of pH_extra_ on H_2_O_2_ production

In agreement with the observations of Selivanov (Selivanov et al. 2008), in non-phosphorylating mitochondria, the acidification of the mitochondrial matrix is followed by an elevation in ΔpH and a decrease in the succinate- and α-GP-driven H_2_O_2_ production. There is an inverse proportionality between ΔpH and H_2_O_2_ formation, which weakens the notion of Lambert and Brand that ΔpH would exhibit a stronger effect on RET than Δψ_m_ (Lambert and Brand 2004). Our measurements of pH_in_ with BCECF have shown that ΔpH is greater at lower pH and varies with pH_extra_. Banh and Treberg observed an analogous pattern in glutamate and malate-respiring, non-phosphorylating, rat skeletal muscle mitochondria, where the H_2_O_2_ generation was enhanced upon alkalization (Banh and Treberg 2013).

### What mechanisms are behind the effects of Δψ_m_ and ΔpH on mitochondrial H_2_O_2_ production?

To understand the effects of Δψ_m_ and ΔpH on the RET-evoked H_2_O_2_ generation, we need to be aware of the production of superoxide (O_2_^**.-**^) by the CI. CI predominantly generates O_2_^**.-**^ (Ohnishi et al. 2005; Grivennikova and Vinogradov 2006). Two mechanistic models exist for the explanation of mtROS production by the CI: (1) the one-site model states that the O_2_^**.-**^ production site, during both FET and RET, is ultimately the reduced flavin (Galkin and Brandt 2005; Pryde and Hirst 2011), whereas (2) the two-site model suggests that during FET, the flavin of CI is responsible for O_2_^**.-**^ formation, while, under RET, the SQ^**.-**^ species, synthetized at the ubiquinone-binding Q-site (Q-binding site) of CI, are liable for the elevated O_2_^**.-**^ release (Brand 2010; Treberg et al. 2011). Both theories agree that the greatest drop in redox potential in the CI occurs between the N2 subunit and the ubiquinone (Q), whose interaction initiates conformational changes that are coupled to the proton translocation (Treberg et al. 2011).

*Δψ*_*m*_*:* There are speculations that the above mentioned conformational changes of the CI might also depend on Δψ_m_ (Brandt 2006; Dlaskova et al. 2008). When Δψ_m_ is adequately high, it decelerates the proton pumping activity of the CI, which may favour SQ^**.-**^ formation and hence O_2_^**.-**^ generation.

*ΔpH and pH*_*extra*_*:* Our results do not support the hypothesis that ΔpH would influence the RET-initiated ROS production to a higher degree than Δψ_m_. The theory that tries to explain the influence of absolute pH on the H_2_O_2_ formation assigns a potential role to SQ^**.-**^ formation at the Q-site of the CI (Ohnishi et al. 2005; Treberg et al. 2011). At the Q-site, Q is reduced by a single electron to SQ^**.-**^. SQ^**.-**^ can react further in two possible ways (Selivanov et al. 2008): (1) with a single electron *plus* two H^+^ to form ubiquinol (QH_2_) (SQ^**.-**^ + e^−^ + 2 H^+^ ↔ QH_2_), or (2) with O_2_ to form the highly reactive O_2_^**.-**^ (SQ^−^ + O_2_ ↔ Q + O_2_^**.-**^). At acidic pH, the first reaction is shifted towards QH_2_ formation according to the Le Chatelier’s principle (Selivanov et al. 2008).

### Potential significance of our results: Mild uncoupling

In succinate-respiring mammalian mitochondria, mild uncoupling lowers Δψ_m_ and consequently also the rate of ROS generation (Skulachev 1996; Korshunov et al. 1997; Miwa and Brand 2003). Mild uncoupling is a special condition where oxidative phosphorylation occurs at a relatively higher conductance of the inner mitochondrial membrane, this results in lowered *pmf* and a minor stimulation of respiration (Skulachev 1996; Brand et al. 2004). Our results support the notion that a minor decrease in Δψ_m_ leads to a diminution of the succinate-evoked, RET-initiated H_2_O_2_ release. Uncoupling proteins (UCP; like UCP1–3) and the adenine nucleotide transporter are also involved in mild uncoupling processes (Andreyev et al. 1988; Jezek 2002). Interestingly, O_2_^**.-**^ can activate UCPs in the matrix with the contribution of fatty acids resulting in mild uncoupling (Echtay et al. 2002) and consequently a slower ROS production. Although it is likely that in vivo*,* under physiological conditions*,* ATP synthesis caused depolarisation of Δψ_m_ is sufficient to decrease ROS generation (Votyakova and Reynolds 2001; Starkov and Fiskum 2003), effects on mtROS of mild uncoupling and of Δψ_m_ are possibly relevant to patological states.

In fact, it has been hypothesized that initiation of mild uncoupling might be beneficial in oxidative stress-related diseases characterized by high Δψ_m_ such as in ischemia-reperfusion injury (Kadenbach et al. 2011). This hypothesis has been corroborated by a report showing that under ischemia, succinate can accumulate in mouse heart owing to the reversal of SDH (Chouchani et al. 2014). In reperfusion, SDH returns to oxidize the accumulated succinate and this has been claimed to result in an enhanced RET-mediated mtROS formation (Chouchani et al. 2014).

In summary, data from our laboratory provided evidence that the succinate- or α-GP-evoked, RET-initiated H_2_O_2_ production is more dependent on Δψ_m_ than on ΔpH. Our findings have helped elucidating mechanisms underpinning mtROS production and support consideration of the therapeutic applications of mild uncoupling, which can be initiated by e.g. mitochondria-targeted antioxidants.

## Abbreviations

α-GP: alpha-glycerophosphate
α-GPDH: alpha-glycerophosphate dehydrogenase
CI: complex I (NADH:ubiquinone oxidoreductase)
CII: complex II (succinate dehydrogenase; succinate:coenzyme Q reductase)
CIII: complex III (coenzyme Q:cytochrome c oxidoreductase)
ΔpH: transmembrane pH gradient
Δψ_m_: mitochondrial membrane potential
mtROS: mitochondrial reactive oxygen species
O_2_^**.**-^: superoxide
pH_extra_: extramitochondrial pH
pH_in_: intramitochondrial pH
*pmf*: proton motive force
Q: ubiquinone
QH_2_: ubiquinol
RET: reverse electron transport
ROS: reactive oxygen species
SDH: succinate dehydrogenase, Complex II, CII
SQ^**.**-^: semiquinone
TPP^+^: tetraphenylphosphonium cation
TPP^+^Cl^−^: tetraphenylphosphonium chloride
UCP: uncoupling protein
